# Optimize Prime/Boost Vaccine Strategies: Trained Immunity as a New Player in the Game

**DOI:** 10.3389/fimmu.2021.612747

**Published:** 2021-03-08

**Authors:** Jean-Louis Palgen, Yanis Feraoun, Gaëlle Dzangué-Tchoupou, Candie Joly, Frédéric Martinon, Roger Le Grand, Anne-Sophie Beignon

**Affiliations:** ^1^Center for Immunology of Viral, Auto-immune, Hematological and Bacterial Diseases (IMVA-HB/IDMIT), Université Paris-Saclay, INSERM, CEA, Fontenay-aux-Roses, France; ^2^School of Medical Sciences, Kirby Institute for Infection and Immunity, Cellular Genomics Futures Institute, University of New South Wales, Sydney, NSW, Australia

**Keywords:** trained immunity, innate immune memory, vaccine, prime/boost vaccine strategies, inflammation, immunization

## Abstract

Most vaccines require multiple doses to induce long-lasting protective immunity in a high frequency of vaccines, and to ensure strong both individual and herd immunity. Repetitive immunogenic stimulations not only increase the intensity and durability of adaptive immunity, but also influence its quality. Several vaccine parameters are known to influence adaptive immune responses, including notably the number of immunizations, the delay between them, and the delivery sequence of different recombinant vaccine vectors. Furthermore, the initial effector innate immune response is key to activate and modulate B and T cell responses. Optimization of homologous and heterologous prime/boost vaccination strategies requires a thorough understanding of how vaccination history affects memory B and T cell characteristics. This requires deeper knowledge of how innate cells respond to multiple vaccine encounters. Here, we review how innate cells, more particularly those of the myeloid lineage, sense and respond differently to a 1st and a 2nd vaccine dose, both in an extrinsic and intrinsic manner. On one hand, the presence of primary specific antibodies and memory T cells, whose critical properties change with time after priming, provides a distinct environment for innate cells at the time of re-vaccination. On the other hand, innate cells themselves can exert enhanced intrinsic antimicrobial functions, long after initial stimulation, which is referred to as trained immunity. We discuss the potential of trained innate cells to be game-changers in prime/boost vaccine strategies. Their increased functionality in antigen uptake, antigen presentation, migration, and as cytokine producers, could indeed improve the restimulation of primary memory B and T cells and their differentiation into potent secondary memory cells in response to the boost. A better understanding of trained immunity mechanisms will be highly valuable for harnessing the full potential of trained innate cells, to optimize immunization strategies.

## Introduction

The goal of vaccination is to elicit long-lasting immune memory, in order to mediate protection from infection, or at least to prevent disease in case of exposure to the pathogen. Multiple immunizations are required for most vaccine strategies, to induce efficient protection. However, there are a few exceptions that elicit life-long protective immunity after a single injection. These vaccines represent the *Grail* for vaccinologists. These include vaccines against yellow fever and smallpox, composed of the yellow fever 17D virus strain (YF17D) and vaccinia virus (VACV), respectively. Even though these are live-attenuated vaccines, what makes them so efficient remains to be completely understood. Mimicking their efficacy is a topic of intense research focus, with the aim to develop new efficient vaccines against other pathogens and diseases.

Repeat vaccinations can be necessary to increase the frequency of responders among vaccinees, and to ensure potent individual and herd immunity. It also enhances and modulates individual immune memory, which is the basis for prime/boost vaccine strategies (see Boxes 1, 2).

Box 1 **First/second vaccine dose and prime/boost.**

In the field, one may encounter the term “primary doses,” rather than “boosts,” particularly when the first vaccine injections are close in time to each other. The very first vaccine dose activates naïve T cells, which undergo proliferation, contraction and a differentiation program to develop into primary memory T cells. As soon as the second vaccine dose is administered, when the primary effector response has started to contract, it can actually be called a boost. It does not always mean that the prime was optimal, and the boost might in fact not only restimulate primary memory T cells, but also prime new naïve T cells, although primary memory T cells have an advantage to respond over naïve T cells.

Box 2 **Homologous vs. heterologous prime/boost vaccine strategies.**

Repeated administrations using the very same vaccine, which are called homologous prime/boost, have proven to be very effective for augmenting humoral responses (1, 2). However, they appeared to be relatively less efficient at enhancing cellular immunity, likely because prior immunity to the vaccine tends to impair robust Ag presentation and the generation of appropriate inflammatory signals for T cells. In contrast, in the 90s, in the context of the development of T cell-based vaccines (e.g., against malaria, *Mycobacterium tuberculosis*, and HIV/AIDS), one strategy to overcome this limitation has been the sequential administration of vaccines using different Ag delivery systems. This approach is called heterologous prime/boost. It has proven to be effective at generating high levels of memory T cells in preclinical studies and clinical trials. However it had never been licensed for humans until very recently with the Gam-COVID-Vac (Sputnik V) aginst COVID-19 (3). It combines recombinant live vectors (such as adenovirus (Ad)- or poxvirus-derived vectors), DNA or RNA vaccines, or adjuvanted subunit vaccines (4).

In addition to the vaccine variables well-known to modulate immunity, such as the nature of the vaccine or adjuvant, its dose and its route of injection for instance, other parameters need to be compared and optimized in the case of prime/boost vaccine strategies (5). They include the number of injections, the delay between them and the combination and order of vaccines for heterologous prime/boost. The exact molecular and cellular mechanisms implicated are not fully understood, preventing a full rationale for optimization of these parameters. Thus, they are defined empirically, and the best parameters out of those tested, neither the absolute nor the individual best parameters, are used.

Although the mechanisms of differentiation of primary and secondary B and T cells after prime and boost are getting better understood, several outstanding questions remain (6, 7). Less is known about the evolution of innate responses after a primary and secondary vaccine encounter, which has likely been overlooked. Classically, innate immunity provides a first line of defense against invading pathogens and shapes adaptive immunity, which takes more time to develop (8–10). However, innate responses can differ between prime and boost, because (1) specific antibodies (Abs), and memory T cells influence innate cells upon re-exposure, and (2) innate cells themselves can functionally and intrinsically differ. Most textbooks still describe similar innate responses after one or more stimulations, independently of the immunological history, because of the short life span of responding innate cells, and the lack of known immune memory in the innate compartment. However, recent insights have challenged this paradigm (11–13). A better understanding of the principles of memory development, of B and T cells, without excluding innate cells, will certainly be important for optimization of prime/boost strategies and defining which vaccine is best to use first and second in a regime, and how long the delay should be between immunizations.

## Innate Responses in the Presence of Specific Antibodies and Memory T Cells

The presence of specific primary Abs and memory T cells at the time of re-vaccination provides a distinct environment to innate cells, which modulates their responses.

### Primary Antibodies

Ab concentration and many biophysical and functional features of Abs are determined by the type of vaccine and vaccine strategy used. Features include Ab affinity, isotypes and subclasses, glycosylation profile, and functions like neutralization, and others that depend on Fc-domain interactions with Fc receptors (FcR) (e.g., antibody-dependent cellular cytotoxicity or antibody dependent cellular phagocytosis). These properties also evolve with time and Ag restimulation.

At re-vaccination, innate cells do not sense immunogens of the vaccine as they did at the time of primary vaccination. At first exposure in a naïve host, the vaccine is “free” and detected solely via Pathogen-Associated Molecular Patterns (PAMPs) and Pattern Recognition Receptors (PRRs) expressed by innate cells. Upon re-exposure, vaccine immunogens form immune complexes with primary Abs. They are cleared by FcR-expressing phagocytic cells, they trigger inflammation and it results in the presentation of vaccine-derived epitopes by these innate cells (14, 15).

Consistently, a vaccine-like effect contributing to protection can be observed in the case of Ab-based immunotherapies against infectious diseases. For instance, in a model of retrovirus infection in mice, passive transfer of Abs resulted in long-term protection (16). It required not only Ab neutralizing- but also Fc-functionality, as neutralization alone failed to protect (17). Neutrophils were required. They mediated B cell help and tuned the humoral response (18). Such a vaccine-like effect of Ab infusion has also been observed in non-human primates, where neutralizing Abs induced strong polyfunctional CD4^+^ T cell response against SIV, mediated by Fc-activated dendritic cells (DCs) (19).

### Primary Memory T Cells

Specific memory T cells respond with more strength, are more frequent and react faster, by requiring less activating signals, than their naïve precursor counterparts (20–22). Like Ab responses, T cell responses are modulated by the number of antigen encounters (20), and also evolve over time. Immune memory differentiation is a *continuum*. Primary and secondary memory T cells, as well as early and late memory T cells differ in their frequency, functions (including proliferation, cytokine production and cytotoxicity), and distribution/recirculation. In particular, a subset of memory T cells, called resident memory T cells (TRM), populate barrier tissues (such as the mucosae and skin) and organs. They do not recirculate like other memory T cell subsets, such as central memory and effector memory T cells (23).

TRM are fostered in the tissue where vaccine is delivered, where they act as sentinels. They react more rapidly to secondary vaccine encounter, and participate in the very early local inflammation and modulation of innate cells. The cytokines they produce can catalyze recruitment, or differentially recruit, activate and license innate cells. For example during influenza infection, it was shown that CD4^+^ memory T cells, can increase the production of innate inflammatory cytokines by antigen-presenting cells (APCs) in the lung upon cell-to-cell contact and cognate antigen (Ag) recognition. This early augmented innate responsiveness likely participates in early control of viral replication (24). Similarly, after immunization with attenuated Listeria monocytogenes, recalled memory T cells rapidly activate innate cells, through an IFN-g/CLL3 dependent mechanism (25, 26). Furthermore, after cognate or even non-cognate recognition of Ag, TRM trigger an innate alarm, which dampens infection severity by recruiting neutrophils into the lungs (27), or by activating DC and NK cells in mucosae of the female reproductive tract (28).

## Innate Cells Can Respond Intrinsically Better to Stimuli After Being Trained

In addition to the extrinsic effect provided by specific Abs and recalled memory T cell responses upon Ag re-exposure, innate cells can react differently to restimulation in an intrinsic manner, because of imprinting that might have occurred during a previous inflammatory/infection episode. Innate cells can display memory-like features, brought about by this so-called innate immune training (11).

### Concept and Hallmarks of Trained Immunity

Trained immunity features and mechanisms differ from those of B and T cells memory by the involvement of metabolic and epigenetic reprogramming in innate cells. It provides homologous (29, 30) and more strikingly heterologous protection (i.e., against antigenically unrelated pathogens), mediated by trained innate cells that display enhanced innate effector response upon restimulation long after the initial stimulus of training. Trained cells remain present at least 3 months after being induced (31), while the non-specific effects (NSE) of live vaccines on all-cause morbidity and mortality, which is thought to be partly mediated by trained immunity in addition to bystander activation and cross-reactive TCR and Ab, last longer, for several years (32, 33). The mechanisms of trained immunity maintenance, and waning remain to be fully investigated (for open questions on trained immunity see Box 3).

Box 3 **Outstanding questions on trained immunity based prime/boost vaccines.**

- Which vaccines and adjuvants are capable of inducing trained immunity?
- Do they stimulate hematopoietic stem or progenitors cells, or a subset, directly or indirectly?
- Are there different mechanisms leading to different flavors of innate memory?
- How long does innate memory take to develop?
- How long does innate memory last?
- Do resting trained cells differ immunophenotypically from their naïve counterparts in addition to their epigenetic marks? Do they represent a distinct subset?
- What are the roles of effector and memory B and T cells, and Abs, in the induction and maintenance of innate memory?
- How to best harness trained immunity to optimize prime/boost vaccine strategies?

### Trainable Cells

The first evidence of innate memory in the myeloid compartment was identified in monocytes/macrophages. Trained monocytes and macrophages were described essentially by their ability to more efficiently produce cytokines, especially IL-6 and TNF-a, upon exposure to unrelated stimuli (29, 34–37). Other cells from the myeloid lineage, such as DCs (29, 38, 39) and even neutrophils (40–43), despite their very short life span, were recently reported to display enhanced innate functions long after the initial stimulation. A burgeoning diversity of neutrophil phenotypes and functionalities are being uncovered, with their capacity to act as APCs a current focus of investigation (44). Innate lymphoid cells (45) and NK cells (46–49) can also “remember” previous infection/inflammation. Ag-specific memory NK cell subsets have been described (50–53). Finally, non-immune cells (such as fibroblast, epithelial stem cells, or interstitial stromal cells) can also be trained, and respond more strongly to tissue stress and damage for instance (54, 55).

### Trained Immunity Mechanisms

**Figure 1:**
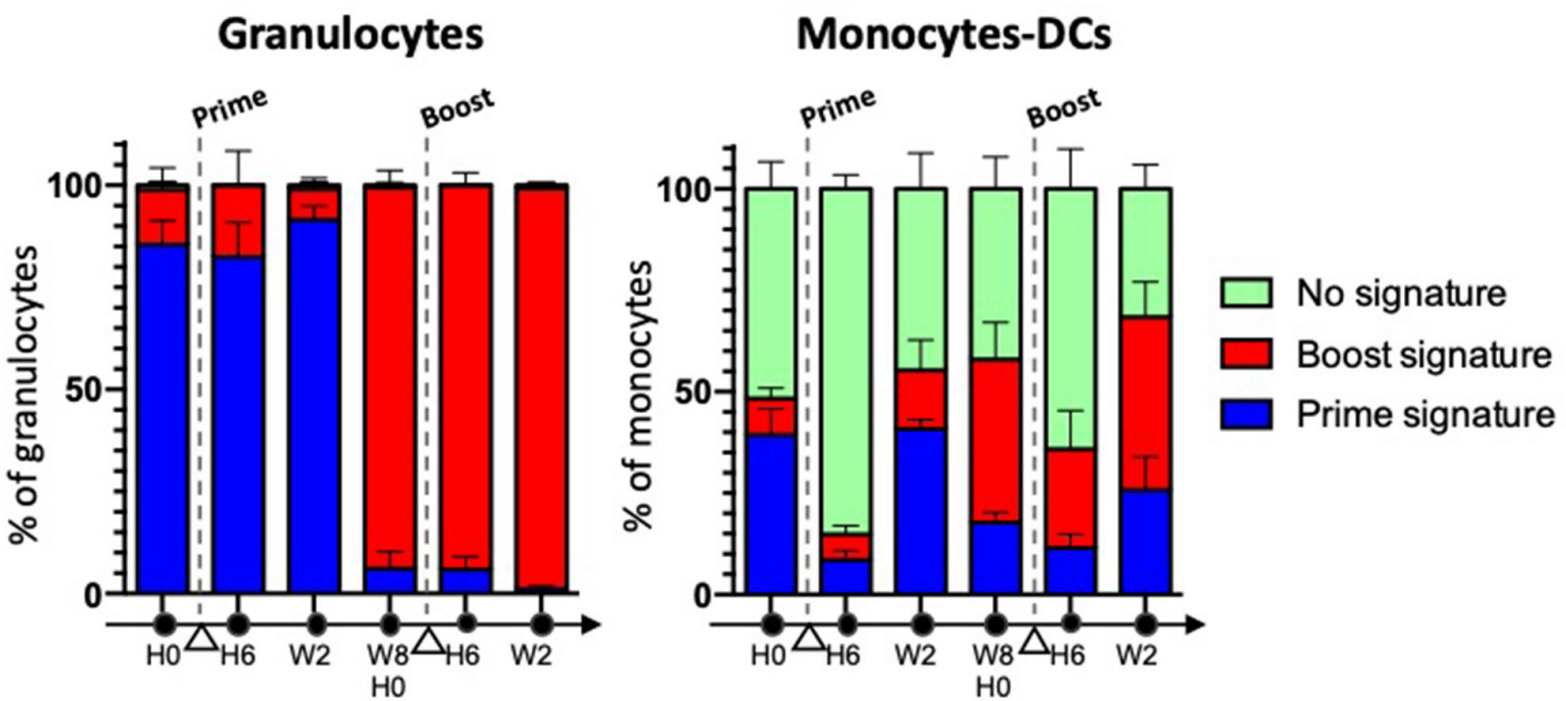
The phenotypic memory of myeloid cells is not restricted to monocytes, and can be more pronounced in granulocytes. Macaques (*n* = 5) were immunized twice, subcutaneously, 2 months apart with a recombinant attenuated vaccinia virus encoding HIV clade B Ags, rec MVA HIV-B (MVA is for Modified Vaccinia Ankara). Blood myeloid cell subsets were analyzed overtime using mass cytometry and a multi-step clustering analysis. They were classified as prime signature (blue), boost signature (red), or non-discriminant “no signature” (green), after a Linear Discriminant Analysis (LDA) performed after Least Absolute Shrinkage and Selection Operator (LASSO). Cell subsets responding to the 2nd immunization differed for the intensity of expression of several markers from those responding to the 1st immunization. They were present prior to vaccine boost, and were induced long after the 1st immunization. They were “better equipped to respond” to restimulation. Most neutrophils were modified, in contrast to some monocytes and DCs (41).

Trained immunity entails the activation, followed by a long-lasting metabolic rewiring, epigenetic re-programming and changes in gene expression in differentiated myeloid cells, such as monocytes (31), and hematopoietic stem and progenitor cells (HSPCs) from the bone marrow (BM), as demonstrated *in vivo* using Bacillus Calmette-Guérin (BCG), the current live attenuated vaccine made of *Mycobacterium bovis* and used against *Mycobacterium tuberculosis* [both in mice (56) and in humans (57)], and with fungal cell wall component b-glucan (58). The transfer of BM cells from BCG- or b-glucan-trained mice into non-trained animals, led to acquisition of trained immunity features in the transplanted animals. Such an education of the progenitors resulted in a bias toward myelopoiesis and was inherited by the myeloid progeny, because epigenetic modifications of HSPCs were stable and durable throughout differentiation. This explains how innate memory can be long-lasting despite the short life of innate effector cells. Myelopoiesis includes several differentiation and maturation steps, which take time, from HSCs to common, and then more committed, myeloid progenitor cells, through to the terminal differentiation of myeloid cells, i.e., granulocytes, monocytes and DCs. Trained daughter innate myeloid cells remain resting when unchallenged and they display enhanced innate effector functions upon stimulation. Differences in the phenotype of resting trained cells and their naïve counterparts has not been explored thoroughly, with the exception of a few studies that demonstrated differential expression of key surface markers between resting trained vs. naïve innate cells (31, 41) (Box 3). In addition, LPS was recently reported to induce long-term cryptic epigenetic changes in *bona fide* hematopoietic stem cells, without modifying their count or gene expression (59). We have previously shown in macaques that the subcutaneous injection of attenuated vaccinia virus, Modified Vaccinia Ankara (MVA), elicited late phenotypic modifications in blood innate myeloid cells resulting in a “defense-ready” phenotype, which was reminiscent of innate training. Monocytes, but also DCs and neutrophils, expressed higher levels of several markers involved in signal transduction (CD45), Ag presentation (HLA-DR), sensing (CD14), binding of immune complexes (CD16, CD32) and complement (CD11b, CD11c), inflammation (IL-10, IP-10, IL-12, IL-8), or migration (CXCR4, CCR5) (41). Admittedly, it remains to be seen whether such phenotypic changes translate into functional innate memory, characterized by an enhanced responsiveness to heterologous stimulation *in vivo*. In any case, our work suggests that MVA imprints different sets of progenitor cells, including downstream of common myeloid progenitors (CMPs)/myeloid-committed granulocyte-monocyte common progenitors (GMPs), because most neutrophils, but only some monocytes and DCs, were modified (Figure 1). GMPs are actually heterogeneous, as committed progenitors within GMPs are now being identified and characterized, as well as their downstream precursors (60–63). Thus, depending on the vaccine and the targeted HSPCs, different flavors of trained immunity are likely to be induced (Box 3).

The development of trained immunity is associated with major HSPC and monocyte changes related to their glycolysis, tricarboxylic acid (TCA) cycle (also called citric acid cycle or Krebs cycle), glutaminolysis, cholesterol synthesis, and fatty acid synthesis, as shown with BCG and b-glucan. Several metabolites, at the intersection of metabolism and epigenetics, are enriched and play key roles in the development and/or persistence of trained immunity. These include fumarate, which accumulates after glutamine replenishment of the TCA, and mevalonate, a metabolite of the cholesterol biosynthesis pathway (36, 58, 64, 65).

Epigenetic reprogramming of HSPCs and monocytes is mediated by histone modifications and deposition of epigenetic marks, in particular H3K4me3 and H3K27Ac marks, on multiple specific targeted loci, i.e. at the promoters and associated enhancers of immune genes (such as PI3K/AKT and NF-kB pathways, as well as TNF-a and IL-6 promoter regions). Whether other epigenetic marks also participate to the regulation of trained immunity needs to be addressed. In addition to histone modification, a role of DNA methylation in the development of trained immunity has also been reported after BCG immunization (66). Some long non-coding RNAs, called immune priming lncRNAs, also play a key role. They are upregulated by the initial stimulus and they direct epigenetic remodeling enzymes proximal to immune genes, and thus target the deposition of epigenetic marks on specific gene promoters (67).

In addition to HSPCs and circulating myeloid cells, trained immunity can be induced locally, as demonstrated in the instance of alveolar macrophages after intranasal infection with non-replicative human serotype 5 adenovirus (Ad5), independently of monocytes and BM HSPCs (68). The training of these macrophages was dependent on IFN-g, produced by effector CD8^+^ T cells, and lasted up to 4 months. Increased glycolytic metabolism, modification of transcriptomic profile, and a heightened response to heterologous (bacterial) infection were observed. This work highlights the need to better understand the role of adaptive effector and memory T cells in the induction and maintenance of innate memory (Box 3).

## Trained Immunity-Based Vaccines

### Trained Immunity-Inducing Vaccines

In the last decade, trained immunity has been abundantly reported following BCG vaccination, in humans and mice, and after b-glucan injection in mice (29, 31, 34, 46, 56, 69, 70). Evidenced by epidemiological, pre-clinical and clinical vaccine studies, the occurrence of NSE and/or trained immunity have been witnessed after administration of live-attenuated vaccines other than BCG, including vaccines against smallpox (vaccinia virus), measles, polio (oral live vaccine, but not the inactivated vaccine), yellow fever, and the new live attenuated *M. tuberculosis* candidate vaccine (*MTB*VAC) (71–76).

What about “non-live” vaccines (such as inactivated or subunit vaccines)? Trivalent influenza vaccination has been reported to elicit imprinting in monocytes and DCs for at least 6 months (77), while gamma-irradiated BCG induced trained monocytes *in vitro*, but failed to do so *in vivo*. In contrast, a long-lasting enhanced anti-inflammatory responsiveness was recently reported after exposure to helminth extracts (78) and a live attenuated anti-pertussis vaccine BPZE1 (79). Finally, diphtheria-tetanus-pertussis (DTP) vaccination, as opposed to BCG, was shown to enhance all-cause morbidity and mortality, more particularly in females (80). Which vaccines/adjuvants can induce trained immunity, and how, is currently one of the hottest topics in the field (Box 3). A better understanding of the mechanisms may make it possible in the future, to precisely target the trained immunity metabolic or epigenetic pathways, with pharmacological modulators, to program and tailor immune training, as recently discussed (81). The genetic depletion and pharmacological inhibition of SHIP-1 was shown for instance to improve the b-glucan mediated training of macrophages (82).

Most current licensed vaccines are administered through parenteral routes. They are highly effective for inducing systemic adaptive immune responses, but they are usually poor at eliciting local immunity. In contrast, mucosal vaccines can induce protective specific immunity at the mucosal front line, through which most pathogens enter the body, and to a lower extent systemically (83). Some vaccines, when delivered by mucosal but not parenteral route, have been shown to also induce trained immunity. A recombinant Ad5-based *M. tuberculosis* vaccine expressing the immunodominant *M. tuberculosis* Ag85A, delivered intranasally afforded protection from early stages of pulmonary *M. tuberculosis* infection; it failed to do so when injected intramuscularly. Protection was mediated by trained airway macrophages (both alveolar and interstitial), and independently of the recruitment of blood inflammatory monocytes in lungs (84). Respiratory-mucosal trained immunity-based vaccination may represent a powerful strategy against respiratory infections, such as *M. tuberculosis* and SARS-CoV-2/COVID-19 (85, 86).

BCG, which is injected intradermally in humans, was recently shown, in a prospective double-blind and randomized clinical trial, to protect the elderly from new infections, especially respiratory infections, and increase the responsiveness of their blood cells to unrelated stimuli (87). Several clinical trials to evaluate whether BCG could protect health workers from SARS-CoV2 infection and COVID-19 are ongoing (88). However, a very encouraging retrospective study comparing healthy volunteers vaccinated with BCG in the last 5 years or never before showed that BCG immunization seems to decrease the incidence of sickness (89). In addition, prior BCG vaccination of health workers was associated with a decrease of SARS-CoV2 seroconversion and of incidence of COVID-19 clinical symptoms. In contrast, the history of meningococcal, pneumococcal, or influenza vaccination did not protect against SARS-CoV-2 infection (90). It is of interest to determine whether BCG delivered to the pulmonary system (by endobronchial instillation) can outperform BCG delivered intradermally, in terms of both systemic and local innate training, as it does in terms of protection against *M. tuberculosis* in non-human primates (91).

The exact stimulus of trained immunity is a matter of great debate. Assuming that vaccine immunogen reaches the BM, HSPCs could be stimulated directly (by detecting vaccine-derived PAMPs), or they could be indirectly stimulated by sensing systemic inflammation signals, including growth factors and cytokines such as GM-CSG, M-CSG, G-CSF, IL-1b, IL-6 (Box 3). The route of administration appears to be a key parameter, and not only the nature of the vaccine itself. The vaccine injection site determines vaccine biodistribution, and which are the first immune and non-immune cells sensing the vaccine, and responding to it, and thus the early and transient inflammation. In mice, BCG injected intravenously persisted in BM monocytes (but not in HSPCs) for up to 7 months and trained immunity developed, whereas subcutaneous BCG injection did not lead to the presence of BCG in the BM and failed to elicit training. However, both routes of BCG injection also likely result in different early systemic inflammation. Additionally, early antibiotic treatment showed that the persistence of BCG in BM was actually not required to induce trained immunity, likely at least its initial presence. In non-human primates, BCG injected intravenously was not found in the BM 1 month later, and, as determined by the production of cytokines in response to heterologous stimulus by PBMCs, there was no evidence of immune training (92). In humans, the intradermal injection of BCG resulted in trained immunity, and no BCG was found in the BM after 90 days (57). In any case, if initial stimulus persists chronically in an anatomical or cellular reservoir, then long-term imprinting of innate cells might not be related to innate memory, but rather reflect a state of chronic stimulation as discussed recently (85). It is also interesting to note that different BCG strains were sub-cultured historically in different laboratories, yielding genetic diversity with differences in virulence, innate activation, immunogenicity and trained immunity-inducing capability (93).

### Trained Immunity to Improve Prime/Boost Vaccines

**Figure 2:**
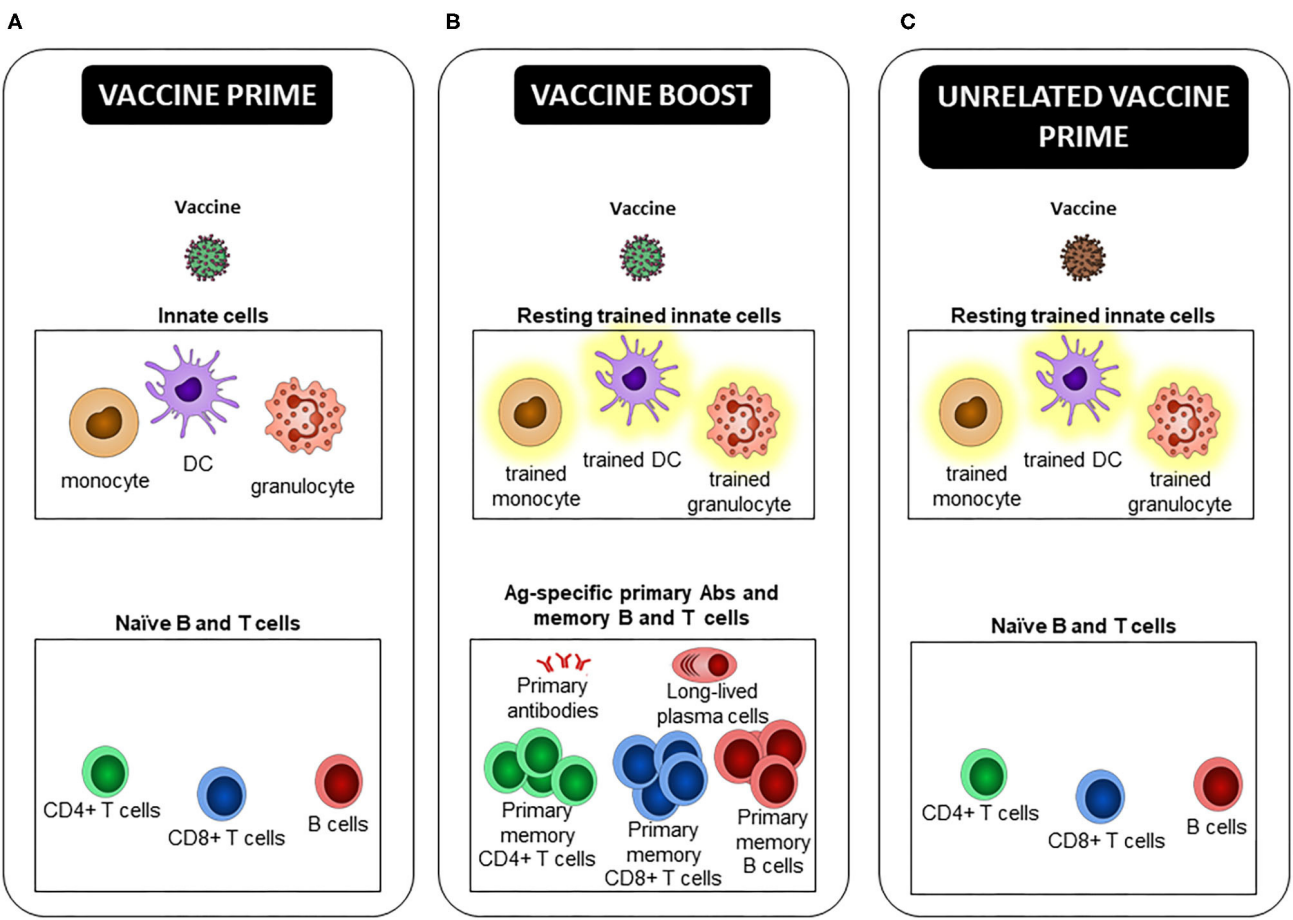
Trained innate immune cells, a new player in prime/boost vaccine strategies. **(A)** At the time of the primary injection, the early innate effector response participates to the activation of Ag specific naive B and T cells, leading to the generation of long-lived plasma cells, primary Abs and memory B and T cells, defined by an Ag-specific heightened effector response upon Ag re-encounter. **(B)** At the time of the vaccine boost, not only “free,” but also Ab-bound vaccine and Ag specific primary memory B and T cells, which are triggered by their cognate Ag recognition, activate innate cells. Depending on the type of vaccine, its route of administration, and the delay between immunizations, prime-induced resting trained innate cells can be present and respond better than “naive” ones to restimulation. These extrinsic, and possibly intrinsic, differences can lead to an innate effector response that differs between prime and boost, and differentially shapes the secondary effector and memory adaptive response. If and how primary Abs, and effector and memory B and T cells participate in the induction and maintenance of trained immunity is unclear. **(C)** In the case of a new unrelated vaccination as opposed to a homologous or heterologous boost, only potential trained HSPCs and innate cells induced by the first vaccine, as well as TRM activated after non-cognate Ag stimulation, may modulate the innate effector response and consequently the primary adaptive response to the second vaccine.

Instead of inducing only classical Ag-specific Ab, B, and T cells, future vaccines could contribute further with induction of both innate and adaptive immune memory as recently highly debated (94–96). Trained immunity-based vaccines could be developed to: (i) increase protection against the targeted infectious agent by relying on both arms of the immune system (innate and adaptive B and T cell responses), (ii) provide heterologous protection against unrelated pathogens, mediated by innate training. More particularly, future vaccines could prevent infection by emerging and old pathogens (such as HIV, RSV, HSV-1/2) for which there remains no potent vaccine. It would benefit more susceptible individuals, such as the newborns and pre-term infants (97), and elderly (87), or patients suffering from immunodeficiency (98). And (iii) pre-condition the innate immune system in order to increase or modulate immune responses after re-vaccination during prime/boost vaccine strategies, or after new unrelated vaccination with a sub-optimal vaccine, or in people less prone to efficiently respond to vaccines, like the elderly (Figure 2).

BCG has been shown to provide innate protection against pathogens and diseases not related to *M. tuberculosis*. It can also potentiate and modulate adaptive immune responses to heterologous pathogens and vaccines. For example, concentrations of specific Abs after routine infant immunization were higher in babies whose innate immune system was exposed to BCG at birth (99). Adults, who were immunized with BCG 2 weeks prior to flu vaccine, developed hemagglutination inhibiting Ab responses, faster and to a greater extent (100). Furthermore, prior immunization with BCG was associated with decreased live-attenuated YF17D vaccine viremia. The BCG-induced lower yellow fever vaccine Ag and PAMP doses had no impact on the yellow fever specific neutralizing Ab response though (69). Thus, suggesting an improved priming, or that YF17D vaccine replication is not a key determinant of the magnitude of the humoral response (likely above a certain concentration), as previously proposed (101).

To benefit from trained immunity in the case of re-vaccination or unrelated vaccination, it is yet to be determined which vaccines, recombinant vectors and adjuvants induce trained immunity, and if specific routes of administration are required. Furthermore, the optimal sequence of immunization needs to be defined, given that the long-term NSE induced by different vaccines can augment/inhibit each other, as demonstrated for BCG and tetanus-diphtheria-pertussis inactivated polio vaccine (Tdap) (102). Therefore, vaccine schedules may need to be adapted. The generation of resting trained innate cells, through the reprogrammation of their HSPCs, takes time. We previously demonstrated in MVA-primed/boosted monkeys that intensity and quality of secondary Ab response correlated with the abundance of trained cells in blood at the time of the 2nd vaccine dose. These cells were not present 2 weeks after the 1st vaccine dose, but were enriched 2 months after (103). Thus, delay between immunizations is another likely key parameter.

## Conclusions

Innate memory is changing our view of vaccines and vaccine strategies. It is a challenging and new tool to improve vaccines. It might also contribute to the inter-individual variability of responses to vaccines, depending on the individual inflammation/infection history that needs to be taken into account to personalize vaccines. Innate training might represent the 6th revolution in vaccinology, next to other breakthroughs such as combination vaccines, new adjuvants, systems vaccinology, and vaccines against non-infectious diseases proposed by Stanley Plotkin (104).

## Author Contributions

J-LP, YF, GD-T, and A-SB wrote the first draft of the manuscript. A-SB oversaw the sections. J-LP and YF designed the figures. CJ, FM, and RL edited the text. All authors discussed the relevant literature and composed the sections of the review article.

## Conflict of Interest

A-SB is the recipient of Sanofi Innovation Award (iAward program), Europe 2020, on Trained Immunity-Inducing Vaccines. The remaining authors declare that the research was conducted in the absence of any commercial or financial relationships that could be construed as a potential conflict of interest.

## References

[1] McShane H Hill A. Prime-boost immunisation strategies for tuberculosis. Microbes Infect. (2005) 7:962–7. 10.1016/j.micinf.2005.03.009 15890555

[2] Ramshaw IA Ramsay AJ. The prime-boost strategy: exciting prospects for improved vaccination. Immunol Today. (2000) 21:163–5. 10.1016/S0167-5699(00)01612-1 10740236

[3] Logunov DY Dolzhikova IV Shcheblyakov DV Tukhvatulin AI Zubkova OV Dzharullaeva AS. Safety and efficacy of an rAd26 and rAd5 vector-based heterologous prime-boost COVID-19 vaccine: an interim analysis of a randomised controlled phase 3 trial in Russia. Lancet. (2021) 397:671–81. 10.1016/S0140-6736(21)00234-8 33545094 PMC7852454

[4] Woodland DL. Jump-starting the immune system: prime-boosting comes of age. Trends Immunol. (2004) 25:98–104. 10.1016/j.it.2003.11.009 15102369

[5] Zimmermann P Curtis N. Factors that influence the immune response to vaccination. Clin Microbiol Rev. (2019) 32:e00084–18. 10.1128/CMR.00084-18 30867162 PMC6431125

[6] McHeyzer-Williams LJ Dufaud C McHeyzer-Williams MG. Do memory B cells form secondary germinal centers? Cold Spring Harb Perspect Biol. (2018) 10:a028878. 10.1101/cshperspect.a028878 28320753 PMC5749153

[7] Masopust D Soerens AG. Tissue-resident T cells and other resident leukocytes. Annu Rev Immunol. (2019) 37:521–46. 10.1146/annurev-immunol-042617-053214 30726153 PMC7175802

[8] Rivera A Siracusa MC Yap GS Gause WC. Innate cell communication kick-starts pathogen-specific immunity. Nat Immunol. (2016) 17:356–63. 10.1038/ni.3375 27002843 PMC4949486

[9] Pulendran B Ahmed R. Immunological mechanisms of vaccination. Nat Immunol. (2011) 12:509–17. 10.1038/ni.2039 21739679 PMC3253344

[10] Sallusto F Lanzavecchia A Araki K Ahmed R. From vaccines to memory and back. Immunity. (2010) 33:451–63. 10.1016/j.immuni.2010.10.008 21029957 PMC3760154

[11] Netea MG Quintin J van der Meer JWM. Trained immunity: a memory for innate host defense. Cell Host Microbe. (2011) 9:355–61. 10.1016/j.chom.2011.04.006 21575907

[12] Aaby P Benn CS Flanagan KL Klein SL Kollmann TR Lynn DJ. The non-specific and sex-differential effects of vaccines. Nat Rev Immunol. (2020) 20:464–70. 10.1038/s41577-020-0338-x 32461674 PMC7252419

[13] O'Sullivan TE Sun JC Lanier LL. Natural killer cell memory. Immunity. (2015) 43:634–45. 10.1016/j.immuni.2015.09.013 26488815 PMC4621966

[14] Bruhns P Jönsson F. Mouse and human FcR effector functions. Immunol Rev. (2015) 268:25–51. 10.1111/imr.12350 26497511

[15] Bournazos S Ravetch JV. Fcγ receptor function and the design of vaccination strategies. Immunity. (2017) 47:224–33. 10.1016/j.immuni.2017.07.009 28813656 PMC5573140

[16] Gros L Dreja H Fiser AL Plays M Pelegrin M Piechaczyk M. Induction of long-term protective antiviral endogenous immune response by short neutralizing monoclonal antibody treatment. J Virol. (2005) 79:6272–80. 10.1128/JVI.79.10.6272-6280.2005 15858011 PMC1091728

[17] Nasser R Pelegrin M Michaud H-A Plays M Piechaczyk M Gros L. Long-lasting protective antiviral immunity induced by passive immunotherapies requires both neutralizing and effector functions of the administered monoclonal antibody. J Virol. (2010) 84:10169–81. 10.1128/JVI.00568-10 20610721 PMC2937798

[18] Naranjo-Gomez M Lambour J Piechaczyk M Pelegrin M. Neutrophils are essential for induction of vaccine-like effects by antiviral monoclonal antibody immunotherapies. JCI Insight. (2018) 3:e97339. 10.1172/jci.insight.97339 29720574 PMC6012508

[19] Yamamoto T Iwamoto N Yamamoto H Tsukamoto T Kuwano T Takeda A. Polyfunctional CD4+ T-cell induction in neutralizing antibody-triggered control of simian immunodeficiency virus infection. J Virol. (2009) 83:5514–24. 10.1128/JVI.00145-09 19297503 PMC2681982

[20] MacLeod MKL Kappler JW Marrack P. Memory CD4 T cells: generation, reactivation and re-assignment. Immunology. (2010) 130:10–5. 10.1111/j.1365-2567.2010.03260.x 20331469 PMC2855788

[21] Veiga-Fernandes H Walter U Bourgeois C McLean A Rocha B. Response of naïve and memory CD8+ T cells to antigen stimulation *in vivo*. Nat Immunol. (2000) 1:47–53. 10.1038/76907 10881174

[22] Wirth TC Xue H-H Rai D Sabel JT Bair T Harty JT. Repetitive antigen stimulation induces stepwise transcriptome diversification but preserves a core signature of memory CD8(+) T cell differentiation. Immunity. (2010) 33:128–40. 10.1016/j.immuni.2010.06.014 20619696 PMC2912220

[23] Paik DH Farber DL. Anti-viral protective capacity of tissue resident memory T cells. Curr Opin Virol. (2020) 46:20–6. 10.1016/j.coviro.2020.09.006 33130326 PMC7979430

[24] Strutt TM McKinstry KK Dibble JP Winchell C Kuang Y Curtis JD. Memory CD4+ T cells induce innate responses independently of pathogen. Nat Med. (2010) 16:558–64, 1p following 564. 10.1038/nm.2142 20436484 PMC2927232

[25] Soudja SM Chandrabos C Yakob E Veenstra M Palliser D Lauvau G. Memory T Cell-derived interferon-γ instructs potent innate cell activation for protective immunity. Immunity. (2014) 40:974–88. 10.1016/j.immuni.2014.05.005 24931122 PMC4105986

[26] Narni-Mancinelli E Soudja SM Crozat K Dalod M Gounon P Geissmann F. Inflammatory monocytes and neutrophils are licensed to kill during memory responses *in vivo*. PLoS Pathog. (2011) 7:e1002457. 10.1371/journal.ppat.1002457 22241983 PMC3248567

[27] Ge C Monk Ir Pizzolla A Wang N Bedford Jg Stinear Tp. Bystander activation of pulmonary trm cells attenuates the severity of bacterial pneumonia by enhancing neutrophil recruitment. Cell Rep. (2019) 29:4236–44.e3. 10.1016/j.celrep.2019.11.103 31875535

[28] Schenkel JM Fraser KA Beura LK Pauken KE Vezys V Masopust D. Resident memory CD8 T cells trigger protective innate and adaptive immune responses. Science. (2014) 346:98–101. 10.1126/science.1254536 25170049 PMC4449618

[29] Quintin J Saeed S Martens JHA Giamarellos-Bourboulis EJ Ifrim DC Logie C. Candida albicans infection affords protection against reinfection *via* functional reprogramming of monocytes. Cell Host Microbe. (2012) 12:223–32. 10.1016/j.chom.2012.06.006 22901542 PMC3864037

[30] Khader SA Divangahi M Hanekom W Hill PC Maeurer M Makar KW. Targeting innate immunity for tuberculosis vaccination. J Clin Invest. (2020) 129:3482–91. 10.1172/JCI128877 31478909 PMC6715374

[31] Kleinnijenhuis J Quintin J Preijers F Joosten LAB Ifrim DC Saeed S. Bacille Calmette-guerin induces NOD2-dependent nonspecific protection from reinfection via epigenetic reprogramming of monocytes. Proc Natl Acad Sci. (2012) 109:17537–42. 10.1073/pnas.1202870109 22988082 PMC3491454

[32] de Bree LCJ Koeken VACM Joosten LAB Aaby P Benn CS. Non-specific effects of vaccines: current evidence and potential implications. Semin Immunol. (2018) 39:35–43. 10.1016/j.smim.2018.06.002 30007489

[33] Balz K Trassl L Härtel V Nelson PP Skevaki C. Virus-induced T cell-mediated heterologous immunity and vaccine development. Front Immunol. (2020) 11:513. 10.3389/fimmu.2020.00513 32296430 PMC7137989

[34] Ciarlo E Heinonen T Théroude C Asgari F Le Roy D Netea MG. Trained immunity confers broad-spectrum protection against bacterial infections. J Infect Dis. (2020) 222:1869–881. 10.1093/infdis/jiz692 31889191 PMC7653089

[35] Garcia-Valtanen P Guzman-Genuino RM Williams DL Hayball JD Diener KR. Evaluation of trained immunity by β-1, 3 (d)-glucan on murine monocytes *in vitro* and duration of response *in vivo*. Immunol Cell Biol. (2017) 95:601–10. 10.1038/icb.2017.13 28228641 PMC5550561

[36] Bekkering S Blok BA Joosten LAB Riksen NP van Crevel R Netea MG. *In vitro* experimental model of trained innate immunity in human primary monocytes. Clin Vaccine Immunol. (2016) 23:926–33. 10.1128/CVI.00349-16 27733422 PMC5139603

[37] Rizzetto L Ifrim DC Moretti S Tocci N Cheng S-C Quintin J. Fungal chitin induces trained immunity in human monocytes during cross-talk of the host with *Saccharomyces cerevisiae*. J Biol Chem. (2016) 291:7961–72. 10.1074/jbc.M115.699645 26887946 PMC4825003

[38] Hole CR Wager CML Castro-Lopez N Campuzano A Cai H Wozniak KL. Induction of memory-like dendritic cell responses *in vivo*. Nat Commun. (2019) 10:2955. 10.1038/s41467-019-10486-5 31273203 PMC6609631

[39] Eastman AJ Xu J Bermik J Potchen N Dekker A Neal LM. Epigenetic stabilization of DC and DC precursor classical activation by TNFα contributes to protective T cell polarization. Sci Adv. (2019) 5:eaaw9051. 10.1126/sciadv.aaw9051 31840058 PMC6892624

[40] Musich T Rahman MA Mohanram V Miller-Novak L Demberg T Venzon DJ. Neutrophil vaccination dynamics and their capacity to mediate B cell help in rhesus macaques. J Immunol. (2018) 201:2287–307. 10.4049/jimmunol.1800677 30217830 PMC6179953

[41] Palgen J-L Tchitchek N Elhmouzi-Younes J Delandre S Namet I Rosenbaum P. Prime and boost vaccination elicit a distinct innate myeloid cell immune response. Sci Rep. (2018) 8:3087. 10.1038/s41598-018-21222-2 29449630 PMC5814452

[42] Kalafati L Kourtzelis I Schulte-Schrepping J Li X Hatzioannou A Grinenko T. Innate immune training of granulopoiesis promotes anti-tumor activity. Cell. (2020) 183:771–85.e12. 10.1016/j.cell.2020.09.058 33125892 PMC7599076

[43] Moorlag SJCFM Rodriguez-Rosales YA Gillard J Fanucchi S Theunissen K Novakovic B. BCG vaccination induces long-term functional reprogramming of human neutrophils. Cell Rep. (2020) 33:108387. 10.1016/j.celrep.2020.108387 33207187 PMC7672522

[44] Lin A Loré K. Granulocytes: new members of the antigen-presenting cell family. Front Immunol. (2017) 8:1781. 10.3389/fimmu.2017.01781 29321780 PMC5732227

[45] Martinez-Gonzalez I Mathä L Steer CA Ghaedi M Poon GFT Takei F. Allergen-experienced group 2 innate lymphoid cells acquire memory-like properties and enhance allergic lung inflammation. Immunity. (2016) 45:198–208. 10.1016/j.immuni.2016.06.017 27421705

[46] Kleinnijenhuis J Quintin J Preijers F Joosten LAB Jacobs C Xavier RJ. BCG-induced trained immunity in NK cells: role for non-specific protection to infection. Clin Immunol. (2014) 155:213–9. 10.1016/j.clim.2014.10.005 25451159 PMC5084088

[47] Schlums H Cichocki F Tesi B Theorell J Beziat V Holmes TD. Cytomegalovirus infection drives adaptive epigenetic diversification of NK cells with altered signaling and effector function. Immunity. (2015) 42:443. 10.1016/j.immuni.2015.02.008 25786176 PMC4612277

[48] Gamliel M Goldman-Wohl D Isaacson B Gur C Stein N Yamin R. Trained memory of human uterine NK cells enhances their function in subsequent pregnancies. Immunity. (2018) 48:951–62.e5. 10.1016/j.immuni.2018.03.030 29768178

[49] Palgen J-L Tchitchek N Huot N Elhmouzi-Younes J Lefebvre C Rosenbaum P. NK cell immune responses differ after prime and boost vaccination. J Leukoc Biol. (2019) 105:1055–73. 10.1002/JLB.4A1018-391RR 30794328

[50] Paust S Blish CA Reeves RK. Redefining memory: building the case for adaptive NK cells. J Virol. (2017) 91:e169–17. 10.1128/JVI.00169-17 28794018 PMC5625515

[51] Adams NM Grassmann S Sun JC. Clonal expansion of innate and adaptive lymphocytes. Nat Rev Immunol. (2020) 20:694–707. 10.1038/s41577-020-0307-4 32424244 PMC13119617

[52] Min-Oo G Kamimura Y Hendricks DW Nabekura T Lanier LL. NK cells: walking three paths down memory lane. Trends Immunol. (2013) 34:251–8. 10.1016/j.it.2013.02.005 23499559 PMC3674190

[53] Geiger TL Sun JC. Development and maturation of natural killer cells. Curr Opin Immunol. (2016) 39:82–9. 10.1016/j.coi.2016.01.007 26845614 PMC4801705

[54] Hamada A Torre C Drancourt M Ghigo E. Trained immunity carried by non-immune cells. Front Microbiol. (2019) 9:3225. 10.3389/fmicb.2018.03225 30692968 PMC6340064

[55] Ordovas-Montanes J Beyaz S Rakoff-Nahoum S Shalek AK. Distribution and storage of inflammatory memory in barrier tissues. Nat Rev Immunol. (2020) 20:308–20. 10.1038/s41577-019-0263-z 32015472 PMC7547402

[56] Kaufmann E Sanz J Dunn JL Khan N Mendonça LE Pacis A. BCG educates hematopoietic stem cells to generate protective innate immunity against tuberculosis. Cell. (2018) 172:176–90.e19. 10.1016/j.cell.2017.12.031 29328912

[57] Cirovic B de Bree LCJ Groh L Blok BA Chan J van der Velden WJFM. BCG vaccination in humans elicits trained immunity via the hematopoietic progenitor compartment. Cell Host Microbe. (2020) 28:322–34.e5. 10.1016/j.chom.2020.05.014 32544459 PMC7295478

[58] Mitroulis I Ruppova K Wang B Chen L-S Grzybek M Grinenko T. Modulation of myelopoiesis progenitors is an integral component of trained immunity. Cell. (2018) 172:147–61.e12. 10.1016/j.cell.2017.11.034 29328910 PMC5766828

[59] de Laval B Maurizio J Kandalla PK Brisou G Simonnet L Huber C. C/EBPβ-dependent epigenetic memory induces trained immunity in hematopoietic stem cells. Cell Stem Cell. (2020) 26:657–74.e8. 10.1016/j.stem.2020.01.017 32169166

[60] Zhu YP Padgett L Dinh HQ Marcovecchio P Blatchley A Wu R. Identification of an early unipotent neutrophil progenitor with pro-tumoral activity in mouse and human bone marrow. Cell Rep. (2018) 24:2329–41.e8. 10.1016/j.celrep.2018.07.097 30157427 PMC6542273

[61] Kwok I Becht E Xia Y Ng M Teh YC Tan L. Combinatorial single-cell analyses of granulocyte-monocyte progenitor heterogeneity reveals an early uni-potent neutrophil progenitor. Immunity. (2020) 53:303–18.e5. 10.1016/j.immuni.2020.06.005 32579887

[62] Evrard M Kwok Iwh Chong Sz Teng Kww Becht E Chen J. Developmental analysis of bone marrow neutrophils reveals populations specialized in expansion, trafficking, and effector functions. Immunity. (2018) 48:364–79.e8. 10.1016/j.immuni.2018.02.002 29466759

[63] Kawamura S Onai N Miya F Sato T Tsunoda T Kurabayashi K. Identification of a human clonogenic progenitor with strict monocyte differentiation potential: a counterpart of mouse cMoPs. Immunity. (2017) 46:835–48.e4. 10.1016/j.immuni.2017.04.019 28514689

[64] Cheng S-C Quintin J Cramer RA Shepardson KM Saeed S Kumar V. mTOR- and HIF-1α-mediated aerobic glycolysis as metabolic basis for trained immunity. Science. (2014) 345:e1250684. 10.1126/science.1250684 PMC4226238 25258083

[65] Arts RJW Novakovic B ter Horst R Carvalho A Bekkering S Lachmandas E. Glutaminolysis and fumarate accumulation integrate immunometabolic and epigenetic programs in trained immunity. Cell Metab. (2016) 24:807–19. 10.1016/j.cmet.2016.10.008 27866838 PMC5742541

[66] Verma D Parasa VR Raffetseder J Martis M Mehta RB Netea M. Anti-mycobacterial activity correlates with altered DNA methylation pattern in immune cells from BCG-vaccinated subjects. Sci Rep. (2017) 7:12305. 10.1038/s41598-017-12110-2 28951586 PMC5615063

[67] Fok ET Davignon L Fanucchi S Mhlanga MM. The lncRNA connection between cellular metabolism and epigenetics in trained immunity. Front Immunol. (2019) 9:3184. 10.3389/fimmu.2018.03184 30761161 PMC6361822

[68] Yao Y Jeyanathan M Haddadi S Barra NG Vaseghi-Shanjani M Damjanovic D. Induction of autonomous memory alveolar macrophages requires T cell help and is critical to trained immunity. Cell. (2018) 175:1634–50.e17. 10.1016/j.cell.2018.09.042 30433869

[69] Arts RJW Moorlag SJCFM Novakovic B Li Y Wang S-Y Oosting M. BCG vaccination protects against experimental viral infection in humans through the induction of cytokines associated with trained immunity. Cell Host Microbe. (2018) 23:89–100.e5. 10.1016/j.chom.2017.12.010 29324233

[70] Kleinnijenhuis J Quintin J Preijers F Benn CS Joosten LAB Jacobs C. Long-lasting effects of BCG vaccination on both heterologous Th1/Th17 responses and innate trained immunity. J Innate Immun. (2014) 6:152–8. 10.1159/000355628 24192057 PMC3944069

[71] Aaby P Martins CL Garly M-L Balé C Andersen A Rodrigues A. Non-specific effects of standard measles vaccine at 4.5 and 9 months of age on childhood mortality: randomised controlled trial. BMJ. (2010) 341:c6495. 10.1136/bmj.c6495 21118875 PMC2994348

[72] Rieckmann A Villumsen M Jensen ML Ravn H da Silva ZJ Sørup S. The effect of smallpox and bacillus calmette-guérin vaccination on the risk of human immunodeficiency virus-1 infection in guinea-bissau and denmark. Open Forum Infect Dis. (2017) 4:ofx130. 10.1093/ofid/ofx130 28852677 PMC5569962

[73] Kölmel KF Grange JM Krone B Mastrangelo G Rossi CR Henz BM. Prior immunisation of patients with malignant melanoma with vaccinia or BCG is associated with better survival. An European Organization for Research and Treatment of Cancer cohort study on 542 patients. Eur J Cancer. (2005) 41:118–25. 10.1016/j.ejca.2004.09.023 15617996

[74] Upfill-Brown A Taniuchi M Platts-Mills JA Kirkpatrick B Burgess SL Oberste MS. Nonspecific effects of oral polio vaccine on diarrheal burden and etiology among bangladeshi infants. Clin Infect Dis. (2017) 65:414–9. 10.1093/cid/cix354 28444240 PMC5848225

[75] Lund N Andersen A Hansen ASK Jepsen FS Barbosa A Biering-Sørensen S. The effect of oral polio vaccine at birth on infant mortality: a randomized trial. Clin Infect Dis. (2015) 61:1504–11. 10.1093/cid/civ617 26219694 PMC4614411

[76] Tarancón R Domínguez-Andrés J Uranga S Ferreira AV Groh LA Domenech M. New live attenuated tuberculosis vaccine MTBVAC induces trained immunity and confers protection against experimental lethal pneumonia. PLoS Pathog. (2020) 16:e1008404. 10.1371/journal.ppat.1008404 32240273 PMC7117655

[77] Galani IE Klechevsky E Andreakos E. Human and translational immunology in the third millennium: progress, challenges and opportunities. Nat Immunol. (2019) 20:1568–73. 10.1038/s41590-019-0543-6 31745346 PMC10424696

[78] Quinn SM Cunningham K Raverdeau M Walsh RJ Curham L Malara A. Anti-inflammatory trained immunity mediated by helminth products attenuates the induction of T cell-mediated autoimmune disease. Front Immunol. (2019) 10:1109. 10.3389/fimmu.2019.01109 31178861 PMC6537856

[79] Cauchi S Locht C. Non-specific effects of live attenuated pertussis vaccine against heterologous infectious and inflammatory diseases. Front Immunol. (2018) 9:2872. 10.3389/fimmu.2018.02872 30581436 PMC6292865

[80] Aaby P Benn C Nielsen J Lisse IM Rodrigues A Ravn H. Testing the hypothesis that diphtheria-tetanus-pertussis vaccine has negative non-specific and sex-differential effects on child survival in high-mortality countries. BMJ Open. (2012) 2:e000707. 10.1136/bmjopen-2011-000707 22619263 PMC3364456

[81] Domínguez-Andrés J van Crevel R Divangahi M Netea MG. Designing the next generation of vaccines: relevance for future pandemics. mBio. (2020) 11:e02616–20. 10.1128/mBio.02616-20 33443120 PMC8534290

[82] Saz-Leal P Del Fresno C Brandi P Martínez-Cano S Dungan OM Chisholm JD. Targeting SHIP-1 in myeloid cells enhances trained immunity and boosts response to infection. Cell Rep. (2018) 25:1118–26. 10.1016/j.celrep.2018.09.092 30380404 PMC6226423

[83] Lycke N. Recent progress in mucosal vaccine development: potential and limitations. Nat Rev Immunol. (2012) 12:592–605. 10.1038/nri3251 22828912

[84] D'Agostino MR Lai R Afkhami S Khera A Yao Y Vaseghi-Shanjani M. Airway macrophages mediate mucosal vaccine-induced trained innate immunity against *Mycobacterium tuberculosis* in early stages of infection. J Immunol. (2020) 205:2750–62. 10.4049/jimmunol.2000532 32998983

[85] Xing Z Afkhami S Bavananthasivam J Fritz DK D'Agostino MR Vaseghi-Shanjani M. Innate immune memory of tissue-resident macrophages and trained innate immunity: re-vamping vaccine concept and strategies. J Leukoc Biol. (2020) 108:825–34. 10.1002/JLB.4MR0220-446R 32125045

[86] Moreno-Fierros L García-Silva I Rosales-Mendoza S. Development of SARS-CoV-2 vaccines: should we focus on mucosal immunity? Expert Opin Biol Ther. (2020) 20:831–6. 10.1080/14712598.2020.1767062 32380868

[87] Giamarellos-Bourboulis EJ Tsilika M Moorlag S Antonakos N Kotsaki A Domínguez-Andrés J. Activate: randomized clinical trial of BCG vaccination against infection in the elderly. Cell. (2020) 183:315–23.e9. 10.1016/j.cell.2020.08.051 32941801 PMC7462457

[88] Madsen AMR Schaltz-Buchholzer F Benfield T Bjerregaard-Andersen M Dalgaard LS Dam C. Using BCG vaccine to enhance non-specific protection of health care workers during the COVID-19 pandemic: a structured summary of a study protocol for a randomised controlled trial in Denmark. Trials. (2020) 21:799. 10.1186/s13063-020-04714-3 32943115 PMC7495402

[89] Moorlag SJCFM van Deuren RC van Werkhoven CH Jaeger M Debisarun P Taks E. Safety and COVID-19 symptoms in individuals recently vaccinated with BCG: a retrospective cohort study. Cell Rep Med. (2020) 1:100073. 10.1016/j.xcrm.2020.100073 32838341 PMC7405881

[90] Rivas MN Ebinger JE Wu M Sun N Braun J Sobhani K. BCG vaccination history associates with decreased SARS-CoV-2 seroprevalence across a diverse cohort of health care workers. J Clin Invest. (2021) 131:e145157. 10.1172/JCI145157 33211672 PMC7810479

[91] Dijkman K Sombroek CC Vervenne RAW Hofman SO Boot C Remarque EJ. Prevention of tuberculosis infection and disease by local BCG in repeatedly exposed rhesus macaques. Nat Med. (2019) 25:255–62. 10.1038/s41591-018-0319-9 30664782

[92] Darrah PA Zeppa JJ Maiello P Hackney JA Wadsworth MH Hughes TK. Prevention of tuberculosis in macaques after intravenous BCG immunization. Nature. (2020) 577:95–102. 10.1038/s41586-019-1817-8 31894150 PMC7015856

[93] Shann F. Editorial commentary: different strains of bacillus Calmette-Guérin vaccine have very different effects on tuberculosis and on unrelated infections. Clin Infect Dis. (2015) 61:960–2. 10.1093/cid/civ454 26060288 PMC4551012

[94] Sánchez-Ramón S Conejero L Netea MG Sancho D Palomares Ó Subiza JL. Trained immunity-based vaccines: a new paradigm for the development of broad-spectrum anti-infectious formulations. Front Immunol. (2018) 9:2936. 10.3389/fimmu.2018.02936 30619296 PMC6304371

[95] Covián C Fernández-Fierro A Retamal-Díaz A Díaz FE Vasquez AE Lay MK. BCG-induced cross-protection and development of trained immunity: implication for vaccine design. Front Immunol. (2019) 10:2806. 10.3389/fimmu.2019.02806 31849980 PMC6896902

[96] Sui Y Berzofsky JA. Myeloid cell-mediated trained innate immunity in mucosal AIDS vaccine development. Front Immunol. (2020) 11:315. 10.3389/fimmu.2020.00315 32184782 PMC7058986

[97] Levy O Levy O. Ready to benefit from training: heterologous effects of early life immunization. Trans R Soc Trop Med Hyg. (2015) 109:3–4. 10.1093/trstmh/tru185 25573103 PMC4351359

[98] Guevara-Hoyer K Saz-Leal P Diez-Rivero CM Ochoa-Grullón J Fernández-Arquero M Pérez de Diego R. Trained immunity based-vaccines as a prophylactic strategy in common variable immunodeficiency. A proof of concept study. Biomedicines. (2020) 8:203. 10.3390/biomedicines8070203 32660100 PMC7400202

[99] Ritz N Mui M Balloch A Curtis N. Non-specific effect of Bacille Calmette-Guérin vaccine on the immune response to routine immunisations. Vaccine. (2013) 31:3098–103. 10.1016/j.vaccine.2013.03.059 23583897

[100] Leentjens J Kox M Stokman R Gerretsen J Diavatopoulos DA van Crevel R. BCG vaccination enhances the immunogenicity of subsequent influenza vaccination in healthy volunteers: a randomized, placebo-controlled pilot study. J Infect Dis. (2015) 212:1930–8. 10.1093/infdis/jiv332 26071565

[101] Muyanja E Ssemaganda A Ngauv P Cubas R Perrin H Srinivasan D. Immune activation alters cellular and humoral responses to yellow fever 17D vaccine. J Clin Invest. (2014) 124:3147–58. 10.1172/JCI75429 24911151 PMC4071376

[102] Blok BA de Bree LCJ Diavatopoulos DA Langereis JD Joosten LAB Aaby P. Interacting, nonspecific, immunological effects of bacille Calmette-Guérin and tetanus-diphtheria-pertussis inactivated polio vaccinations: an explorative, randomized trial. Clin Infect Dis. (2020) 70:455–63. 10.1093/cid/ciz246 30919883

[103] Palgen J-L Tchitchek N Rodriguez-Pozo A Jouhault Q Abdelhouahab H Dereuddre-Bosquet N. Innate and secondary humoral responses are improved by increasing the time between MVA vaccine immunizations. npj Vaccines. (2020) 5:1–16. 10.1038/s41541-020-0175-8 PMC7081268 32218996

[104] Plotkin SA. Six revolutions in vaccinology. Pediatr Infect Dis J. (2005) 24:1–9. 10.1097/01.inf.0000148933.08301.02 15665703

